# Supplementary material for: Evolutionary Wheat Populations in High-Quality Breadmaking as a Tool to Preserve Agri-Food Biodiversity
Source: Foods. 2022 Feb 9;11(4):495. doi: 10.3390/foods11040495 (PMC8871435; doi:10.3390/foods11040495)
Supplement: Supplementary file 1 [file foods-11-00495-s001.zip › foods-1554429-supplementary-done.pdf]

foods

Article

Evolutionary wheat populations in high-quality breadmaking as a tool to preserve agro-food biodiversity

Marco Spaggiari[1], Mia Marchini[2], Luca Calani[1], Rossella Dodi[3], Giuseppe Di Pede[1], Margherita Dall'Asta[4], Francesca Scazzina[1], Andrea Barbieri[5], Laura Righetti[1], Silvia Folloni[2,*], Roberto Ranieri[2], Chiara Dall'Asta[1], Gianni Galaverna[1]

[1] Department of Food and Drug, University of Parma, Parco Area delle Scienze 17/A, 43124 Parma, Italy; marco.spaggiari1@studenti.unipr.it (M.S.); luca.calani@unipr.it (L.C.); giuseppe.dipede@unipr.it (G.D.P.); francesca.scazzina@unipr.it (F.S.); laura.righetti@unipr.it (L.R.); chiara.dallasta@unipr.it (C.D.); gianni.galaverna@unipr.it (G.G.).
[2] Open Fields s.r.l., str. Madonna dell'Aiuto 7/A, Parma, Italy; m.marchini@openfields.it (M.M.); r.ranieri@openfields.it (R.R.).
[3] Department of Veterinary Science, University of Parma, Strada del Taglio 10, 43126 Parma, Italy; rossella.dodi@unipr.it (R.D.).
[4] Faculty of Agriculture, Food and Environmental Sciences, Catholic University of the Sacred Heart, via Emilia Parmense 84, 29122 Piacenza, Italy; margherita.dallasta@unicatt.it (M.D).
[5] Molino Grassi SpA, via Emilia ovest 347, 43126 Fraore Parma, Italy; andreabarbieri@molinograssi.it (A.B.).
* Correspondence: s.folloni@openfields.it (S.F.); Tel.: +39 05211812730)

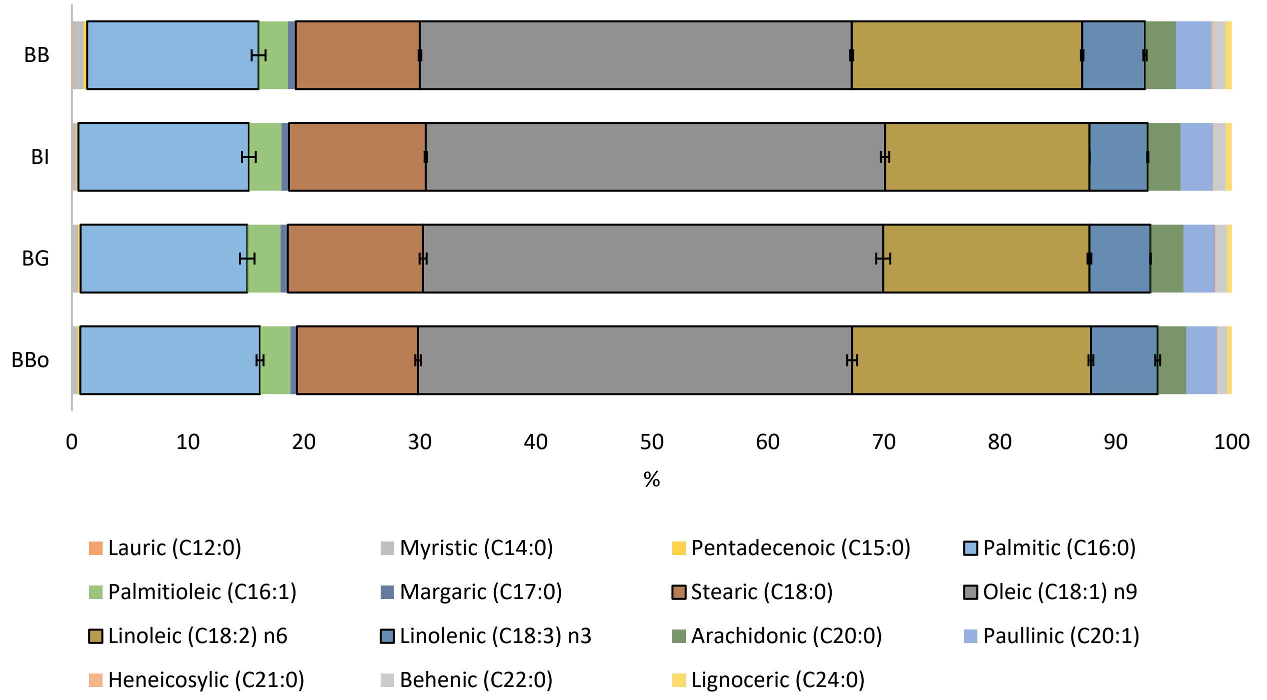

Figure S1 Fatty acids (FAs) profile of the different breads. Results are reported as cumulative percentage (%) of FAs. BB, bread produced using BIO2 EP; BI, bread produced using ICARDA EP; BG, bread produced using Grossi EP; BBo, bread produced using cv. Bologna.

Table S1 Mass spectrometry characteristics of nicotinamide, nicotinic acid, thiamine, and folic acid.

Compound	CID	Monitored ion	Monitored transitions *
		$[M+H]^+$	*(m/z)*
Nicotinamide	40	123	123 → **80**; 123 → 95; 123 → 105; 123 → 122
Nicotinic acid	37	124	124 → **80**; 124 → 123; 124 → 105
Thiamine	22	265	265 → **122**; 265 → 144
Folic acid	17	442	442 → **295**; 442 → 313

CID: Collision Induced Dissociation. * The quantifier ion (*m/z*) is reported in bold.

Table S2 Standard deviation (n=3) of the grain quality parameters of EPs and cv. Bologna.

Wheat	Test weight (kg/hL)	Thousand kernel weight (g)	Protein content (% d.m.)	Alveograph			
				W (10^{-4}J)	P (mm H_2O)	L (mm)	P/L
Bio2 EP	1	0	1.28	13.50	1.5	9	0.07
ICARDA EP	3	0	1.72	8.50	2	11.5	0.06
Grossi EP	2	1	0.53	8.50	1	16.5	0.02
Bologna	0	1	0.40	4.00	1.5	0.5	0.05

EP, evolutionary wheat population.

Table S3 Standard deviation (n=3) of the nutritional and chemical composition of the bread formulated using the wheat evolutionary population (BB, BI and BG) and bread produced using flour from cv. Bologna wheat (BBo).

	BB	BI	BG	BBo
Carbohydrates (g/100 g)	1.2	1.1	1.3	1.4
Total dietary fibre (g/100g)	0.94	0.89	0.95	1.03
Lipids (g/100g)	0.01	0.03	0.03	0.07
SFA (%)	0.4	0.1	0.8	0.6
MUFA (%)	0.2	0.2	0.6	0.6
PUFA (%)	0.3	0.1	0.2	0.1
Ω-6 / Ω-9	0.1	0.1	0.0	0.2
Proteins (g/100g)	1.3	1.7	1.1	1.6
Mg (mg/100g)	2.5	2.2	2.1	3.2
Zn (mg/100g)	0.17	0.15	0.16	0.16
Fe (mg/100g)	0.21	0.13	0.17	0.21
Se (µg/100g)	0.20	0.20	0.22	0.22
Thiamine (mg/100g)	0.01	0.01	0.01	0.12

Nicotinic acid (mg/100g)	<LOQ	<LOQ	<LOQ	<LOQ
Folic acid (μg /100g)	<LOQ	<LOQ	<LOQ	<LOQ
Nicotinamide (mg/100g)	0.01	0.11	0.01	0.10

<LOQ Folic acid: 5 μg/100 g; <LOQ Nicotinic acid: 0.01 mg/100 mg, Mg, magnesium; Zn, zinc; Fe, iron; Se, selenium; NAM, nicotinamide; BB, bread produced using BIO2 EP; BI, bread produced using ICARDA EP; BG, bread produced using Grossi EP; BBo, bread produced using cv. Bologna. *: Calories (kJ and kcal) were calculated as sum of nutritive components.

Table S4 Standard deviation (n=3) of the micronutrients content in flours

	**FB**	**FI**	**FG**	**FBo**
Mg (mg/100g) *	2.9	2.6	2.9	4.4
Zn (mg/100g) *	0.23	0.19	0.22	0.24
Fe (mg/100g) *	0.27	0.13	0.27	0.28
Se (μg /100g) **	0.68	0.62	0.85	0.99
Thiamine (mg/100g) *	0.01	0.00	0.08	0.03
Nicotinic acid (mg/100g) *	<LOQ	<LOQ	<LOQ	<LOQ
Nicotinamide (mg/100g) *	0.03	<LOQ	0.08	0.01
Folic acid (μg /100g) **	4.4	<LOQ	<LOQ	<LOQ

* <LOQ, 0.01 mg/100g; ** <LOQ, 0.5 μg/100 g. FB, BIO2 EP Type 1 flour; FI, ICARDA EP Type 1 flour; FG, Grossi EP Type 1 flour; FBo, cv. Bologna Type 1 flour.

Table S5 Standard deviation (n=3) of the total phenolic content (TPC) and phenolic acid (PA) profile in their free (soluble) and bound (insoluble) forms.

**Sample**	**TPC**		**4-HB**		**p-C**		**Caff**		**t-Fer**		**c-Fer**	**Sin**	
	**Free**	**Bound**	**Free**	**Bound**	**Free**	**Bound**	**Free**	**Bound**	**Free**	**Bound**		**Free**	**Bound**
**Flours**	mg GAE/Kg d. m.							mg/Kg d.w.					
FB	23.98	92.15	0.01	<LOQ	<LOQ	0.01	0.0	0.03	0.06	0.12	0.08	0.04	0.11
FI	13.83	38.67	0.01	<LOQ	0.01	0.01	0.01	0.01	0.05	0.15	0.02	0.01	0.03
FG	12.73	64.37	<LOQ	<LOQ	0.01	0.01	0.02	0.01	0.13	0.16	0.15	0.03	0.07
FBo	9.60	41.65	<LOQ	<LOQ	<LOQ	0.03	0.02	0.01	0.14	0.20	0.01	0.01	0.1
**Breads**													
BB	35.43	33.41	<LOQ	0.04	<LOQ	0.01	<LOQ	0.04	0.3	0.25	4.11	<LOQ	0.15
BI	13.86	3.06	<LOQ	0.1	<LOQ	0.11	<LOQ	0.01	0.21	3.00	8.23	0.11	0.07
BG	8.07	4.05	<LOQ	0.1	0.04	0.07	<LOQ	0.01	0.34	1.46	1.79	0.04	0.47
BBo	0.36	16.55	0.04	0.15	0.01	0.51	<LOQ	0.15	0.20	9.55	2.49	<LOQ	1.19

<LOQ: 0.05 mg/kg. GAE, Gallic Acid Equivalents; d. m., dry matter; 4-HB, hydroxybenzoic acid; p-C, para coumaric acid; caff, caffeic acid; *t*-fer, *trans*-ferulic acid; *c*-fer, *cis*-ferulic acid; Sin, sinapic acid. FB, BIO2 EP Type 1 flour; FI, ICARDA EP Type 1 flour; FG, Grossi EP Type 1 flour; FBo, cv. Bologna Type 1 flour; BB, bread produced using BIO2 EP; BI, bread produced using ICARDA EP; BG, bread produced using Grossi EP; BBo, bread produced using cv. Bologna.

Table S6 Standard deviation (n=59) of the sensory scores of breads obtained from acceptability test.

Bread	Texture		Colour		Appearance	Aroma	Taste	Overall acceptability
	Crust	Crumb	Crust	Crumb				
**BI**	1.54	1.36	1.58	1.39	1.14	1.30	1.50	1.25
**BB**	1.52	1.10	1.23	1.19	1.19	1.24	1.39	1.03
**BG**	1.52	1.29	1.50	1.09	1.13	1.17	1.48	1.23
**BBo**	1.75	1.55	1.46	1.89	1.59	1.39	1.66	1.41

BB, bread produced using BIO2 EP; BI, bread produced using ICARDA EP; BG, bread produced using Grossi EP; BBo, bread produced using cv Bologna.

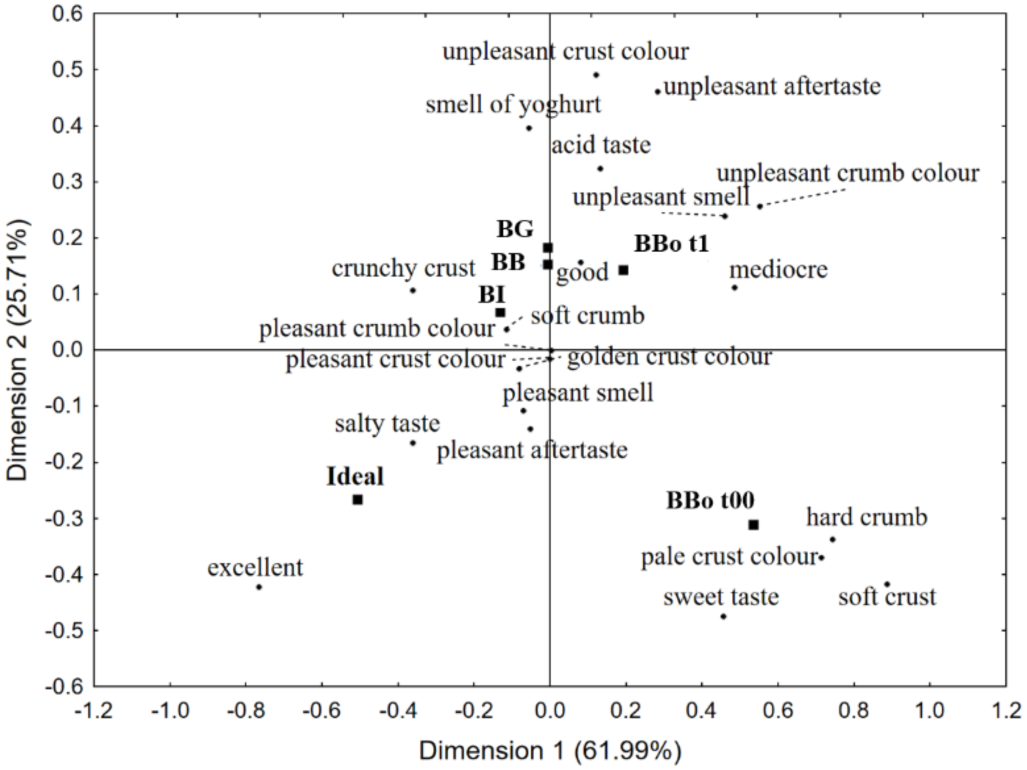

Figure S2 Correspondence analysis of the bread samples and sensory attributes including a Bologna type 00 control bread. BB, bread produced using Bio2 EP; BI, bread produced using ICARDA EP; BG, bread produced using Grossi EP; BBo t00, bread produced using cv. Bologna flour type 00; BBo t1, bread produced using cv. Bologna flour type 1.
